# Comprehensive Iranian guidelines for the diagnosis and management of maple syrup urine disease: an evidence- and consensus- based approach

**DOI:** 10.1186/s13023-025-03533-6

**Published:** 2025-01-07

**Authors:** Noushin Rostampour, Setila Dalili, Hossein Moravej, Zhila Afshar, Negar Yazdani, Seyedeh Tahereh Mousavi, Parastoo Rostami, Daniel Zamanfar, Maryam Yahay, Abdolhossein Nikravesh, Zahra Beyzaei, Mohamad Ahangar Davoodi, Atefeh Sedaghat, Tahora Hakemzadeh, Ali Talea

**Affiliations:** 1 Liver Disease Research Center, Isfahan University of Medical Sciences, Isfahan, Iran; 2 Pediatric Diseases Research Center, Guilan University of Medical Sciences, Rasht, Iran; 3 Neonatal Research Center, Shiraz University of Medical Sciences, Shiraz, Iran; 4 Department of Pediatrics Endocrinology, Shiraz University of Medical Sciences, Shiraz, Iran; 5 Community Based Psychiatric Care Research Center, School of Nursing and Midwifery, Shiraz University of Medical Sciences, Shiraz, Iran; 6 Assisstant Professor of Pediatrics Endocrinology, School of Medicine, Bushehr University of Medical Sciences, Bushehr, Iran; 7 Division of Endocrinology and Metabolism, Department of Pediatrics, Children’s Medical Center, Tehran University of Medical Sciences, Tehran, Iran; 8 Diabetic Research Center, Mazandaran University of Medical Sciences, Mazandaran, Iran; 9 Department of Clinical Nutrition Food Sciences & Technology, School of Nutrition & Food Sciences, Isfahan University of Medical Sciences, Isfahan, Iran; 10 Department of Pediatric Endocrinology & Metabolism, Bushehr University of Medical Sciences, Bushehr, Iran; 11 Shiraz Transplant Research Center (STRC), Shiraz University of Medical Sciences, Shiraz, Iran; 12 Department of Pediatric Endocrinology & Metabolism, Arak University of Medical Sciences, Clinical Research Development Center of Amirkabir Hospital, Arak, Iran; 13 Pediatric Endocrinologist, Metabolic Disorders Research Center, Molecular-cellular Endocrinology & Metabolism Research Institute, Tehran University of medical Sciences, Tehran, Iran

**Keywords:** Maple syrup urine disease, Inherited metabolic disorder, *BCKDHA*, *BCKDHB*

## Abstract

Maple Syrup Urine Disease (MSUD) disease is a defect in the function of the Branched-chain 2-ketoacid dehydrogenase complex (BCKDH). It is caused by pathogenic biallelic variants in BCKDHA, BCKA decarboxylase, or dihydrolipoamide dehydrogenase. The brain is the major organ involved in MSUD. MSUD happens in about 1 in 86,800 to 185,000 live births. According to some diversity in the management of Iranian patients with MSUD, the development of a national guideline is essential. This guideline is provided through a literature search on articles in PubMed, Scopus, Web of Sciences, Cochrane, and Embase databases from 2001 to 2022 accompanied by a consensus of physicians of different centers in Iran who are experts in the diagnosis and management of this disease. This article considers pathogenesis, epidemiology, clinical manifestations, diagnosis, treatment, and monitoring of MSUD patients with limited recourse.

## Background

**Fig. 1:**
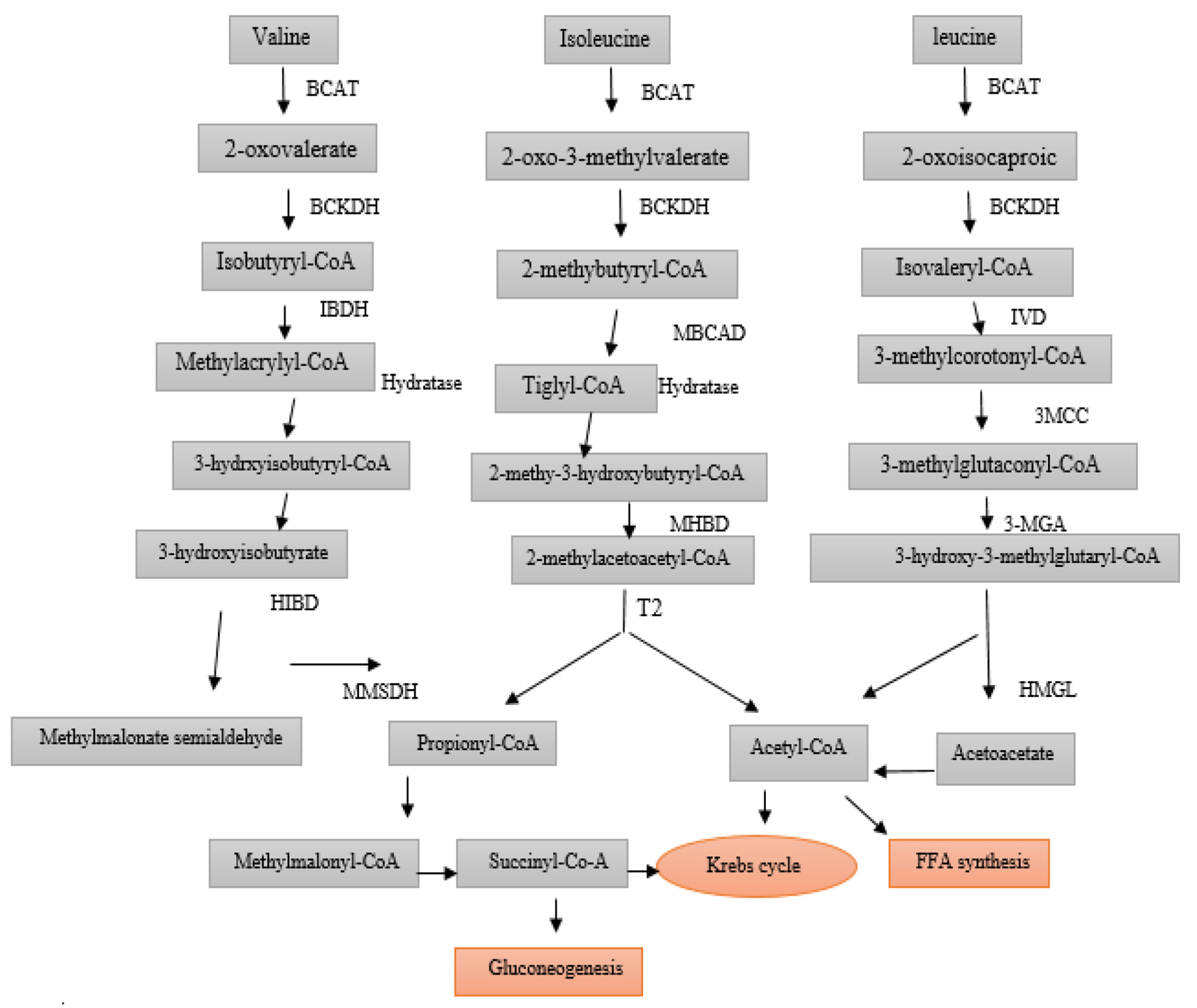
Metabolic Pathway of Branched Chain Aminoacids. BCAT: Branched−chain amino acid transferase, BCKDH: Branched chain alpha−keto acid dehydrogenase, IBDH: Isobutyryl CoA dehydrogenase, MBCAD: Methyl butyryl CoA dehydrogenase, IVD: Isovalery CoA dehydrogenase, 3−MCC: 3−methylcrotonyl−CoA Carboxylase, HIBDA: 3−Hydroxyisobutyryl CoA deacetylase, MHBD: 2−methyl−3−hydroxyisobutyric dehydrogenase, 3−MGA: 3−methylglutaconic−CoA hydratase, HIBDH: 3−Hydroxy isobutyrate dehydrogenase, T2: mitochondrial acetoacetyl−CoA thiolase. HMGL: 3−hydroxy3−methylglutaryl−CoA lyase, MMSDH: Methylmalonic semialdehyde dehydrogenase, PCC: Propionyl coA carboxylase

Branched-chain amino acids (BCAAs) (leucine, isoleucine, and valine) make up about 35% of all amino acids in muscles and are the most important and efficient source of energy [1]. The second enzyme in the metabolism of BCAAs is the Branched-chain 2-ketoacid dehydrogenase complex (BCKDH). In maple syrup urine disease (MSUD) disease (MSUD; MIM 248600), there is a defect in the function of this enzyme (Fig. 1). BCKDH consists of three subunits: E1, E2, and E3 [1]. The brain is the only organ involved in MSUD.

MSUD happens in about 1 in 86,800 to 185,000 live births [2]. The prevalence of this disease is reported to be higher in countries where consanguineous marriage is more common (1 in 50,000 in Turkey) [3]. We do not have accurate information on disease prevalence in Iran. The inheritance pattern of this disease is autosomal recessive. The genes encoding the components of the branched-chain alpha-keto acid dehydrogenase complex (*BCKDH*) E1-α, E1-β, E2, and E3 are located on chromosomes 19q13.1-q13.2 (*BCKDHA*), 6p22-p21 (*BCKDHB*), 1p31 (*DBT*), and 7q31-q32 (*DLD*) respectively. There is no precise genotype-phenotype relationship in MSUD patients [4]. Biochemical disorders caused by pathogenic biallelic variants in *BCKDHA*, which encodes the alpha subunit of BCKA decarboxylase (E1), are known as MSUD type 1 A. A biochemical disorder caused by biallelic pathogenic variants in *BCKDHB*, which encodes the beta subunit of BCKA decarboxylase (E1), is referred to as MSUD type 1B. additionally, biallelic pathogenic variants in *DBT* encoding the dihydrolipoyl transacylase (E2) subunit is also called MSUD type 2. All three are clinically and biochemically indistinguishable. Dihydrolipoamide dehydrogenase (DLD) deficiency, caused by biallelic pathogenic variants in DLD encoding the E3 subunit (lipoamide dehydrogenase) of BCKD, is sometimes referred to as MSUD type 3. However, the phenotype is easily distinguishable from MSUD [5].

Clinical presentation and severity of MSUD varies based on the subtypes. The classic presentation typically occurs during the neonatal period and is characterized by failure to thrive, developmental delays, feeding challenges, and the distinct maple syrup odor in the cerumen and urine. If left untreated, it can result in irreversible neurological complications such as stereotypical movements, metabolic decompensation, and potentially fatal outcomes. Diagnosis involves identifying elevated ratios of (leucine + isoleucine) to alanine and phenylalanine, increased levels of BCAAs, and alloisoleucine. In Iran, MSUD patients are identified through the NBS process leading to identifying more MSUD patients. The NBS was performed in some parts of Iran for 20 *Inborn Errors of Metabolism (IEM).* All newborn neonates are screened for 20 inherited metabolic diseases three to five days after birth. The metabolic screening test is performed using Tandem Mass Spectrometry (MS/MS) method in the reference laboratory and includes some of the amino acids and acylcarnitines tested in dried blood spots (DBS) obtained from the heel prick of the newborns. Confirmation of MSUD diagnosis is based on the detection of biallelic pathogenic variants in the genes BCKDHA, BCKDHB, or DBT. The treatment includes a protein-restricted diet and supplementation with a specialized formula containing essential amino acids (excluding BCAA) and micronutrients to prevent the onset of neurological symptoms.

Access to laboratory facilities and essential therapeutic resources, such as formulas, special food, and pure amino acids, varies in each country. In developing countries, the management and monitoring of MSUD patients pose challenges. These include issues such as amino acid analysis not being reimbursed by insurance, costly and time-consuming processes (taking over a week to get test results), limited availability of supplements (e.g., Valine, Isoleucine), and a lack of patient support services like physiotherapy. Therefore, most countries need specific guidelines that are compatible with their facilities. This article provides a guideline that can be useful for all physicians, primary care providers, and other specialists who often are involved in the care of individuals with MSUD in Iran and other countries with the same situation of laboratory and therapeutic material.

## Methods

This study was conducted through a literature search on articles in English with the keywords MSUD or Maple syrup urine disease in PubMed, Scopus, Web of Sciences, Cochrane, and Embase databases from 2001 to 2022. The cooperation invitation was sent to all physicians in Iran who work in the clinics of inherited metabolic diseases and have had the opportunity to treat patients with MSUD. These physicians have a national board of subspecialty in the field of hereditary metabolic diseases. Physicians who declared their readiness were included as the authors. All related articles were sent to them. Each of the authors was responsible for writing a part of the article. Then the written content was distributed to other authors of the article to comment on it. Disagreements at every part were resolved by consensus and referred back to the original article. Various viewpoints and ideas were exchanged and debated to ensure a comprehensive and evidence-based approach to the recommendations provided.

## Results

### Clinical manifestations of MSUD

MSUD is categorized into two main forms – classic and variants – based on the severity of enzyme deficiency and clinical symptoms. Variants include intermittent, intermediate, thiamine-responsive, and E3 deficiency [5].

Classic MSUD phenotype typically manifests in newborns, whereas, the other variants may present at any time during infancy or childhood, usually triggered by episodes of stress, such as concurrent illness, fasting, or surgery. The patients with classic type of MSUD are normal at birth, but they present clinical manifestation including poor feeding, vomiting, drowsiness, gradually leading to irritability, lethargy, and a decrease in consciousness after breast and formula feeding, in the first weeks of life. The timing of presentation is closely linked to the protein content in their diet. Coma and cerebral edema symptoms occur as bulging of fontanel, pupillary changes, bradycardia, hypothermia, and hypotonia [6]. They may also have mild hepatomegaly with dehydration.

One of the most important features of this disease is hypotonia and hypertonia attack, which occur in the form of boxing/cycling/opisthotonos/ myoclonus movements [7] as well as limb jerks, limb stiffness, and axial hypotonia. These attacks can also occur after infection, change in nutrition, surgery, and any aggravated state of catabolism. During adulthood, symptoms of attacks may present as abdominal pain, vomiting, anorexia or neurological symptoms (dystonia), ataxia, reduced mental function and hemiplegia, hyperactivity, sleep disorders, and encephalopathy attacks. The clear odor of maple syrup can be smelled in classic form or during catabolic attacks [8].

Intermittent MSUD is the second most common type of MSUD. Patients with intermittent MSUD usually present with ketoacidosis during periods of catabolic stress with signs of neurotoxicity. Death of patients with intermittent form may occur without appropriate diagnosis and treatment. Patients with intermediate MSUD may become symptomatic at any age. Most of them are detected between five months and seven years [9]. Intermediate and Thiamine responsive forms are less common. They have moderate and similar clinical manifestations. E3 deficiency is rare. The affected patients have hypotonia, developmental delay, dystonia/chorea, and Leigh-type encephalopathy. Clinical manifestations of all types of MSUD are summarized in Table 1.

**Table 1:** Clinical phenotypes and overview of maple syrup urine disease

Clinical phenotypes	Age of onset^1^	Severity	Acute metabolic decompensation	Response t0 thiamine	Clinical features	Biochemical signs	% Residual BCKD activity^2^
Classic	Newborn	Moderate to severe	Yes (newborn period and with catabolic stress)	No	Maple syrup odor of cerumen Poor feeding Irritability, lethargy Opisthotonus Focal dystonia “Fencing,” “bicycling” Obtundation, coma Central respiratory failure	↑ BCAAs in plasma ↑ plasma alloisoleucine ↑ BCKAs in urine Ketonuria	0-2%
Intermediate	Infancy to childhood	Moderate to severe	Yes (with catabolic stress)	No	Maple syrup odor of cerumen Poor growth Poor feeding Irritability Developmental delays Encephalopathy during illness Neurologic symptoms	Similar to classic phenotype, though quantitatively less severe	3-30%
Intermittent	Infancy to childhood, or adulthood (rare)	Mild to moderate	Rare	No	Normal early growth & development Learning disability Neurological symptoms Episodic decompensations that can be severe	Normal BCAAs when the patient is well similar to the classic biochemical profile during illness	5-20%
Thiamine responsive	Infancy to childhood	Moderate	Rare	In conjunction with dietary therapy	Similar to intermediate phenotype	Improvement of leucine tolerance & biochemical profile with thiamine therapy	2-40%
E3 ²	Newborn	Moderate	rare	No	hypotonia and developmental delay	persistent lactic acidosis with high levels of plasma pyruvate and alanine. Plasma BCAA concentrations are moderately increased.	Not known

^BCAAs: branched−chain amino acids; BCKAs: branched−chain alpha−ketoacidosis^

### Diagnosis

MSUD should be suspected in asymptomatic patients with abnormal neonatal screening and/or symptomatic cases with clinical manifestations suspicious to MSUD. These manifestations include metabolic acidosis with a normal anion gap, convulsion, hypoglycemia refractory to treatment with or without symptoms, hypertonia, boxing& bicycling, and sometimes peculiar odor of maple syrup in urine or body [10]. Hence, to diagnose this disease, various scenarios are considered:

#### Scenario 1: abnormal newborn screening (NBS)

NBS for MSUD is primarily based on assessing the ratios of (leucine + isoleucine) to alanine and phenylalanine concentrations on dried blood spots. If the screening is positive (i.e. leucine + isoleucine > 250 nmol/L or its ratio to alanine and phenylalanine concentrations > 3), the neonate needs confirmatory biochemical testing with measurement of plasma amino acids and alloisoleucine. If BCAAs are significantly elevated and/or alloisoleucine > 2 nmol/ml, treatment should be initiated immediately. It should be noted that leucine, isoleucine, and hydroxyproline cannot be differentiated by mass spectrometry. Therefore, neonates with isolated hydroxyprolinemia have screening results suspicious to MSUD, but confirmatory plasma amino acid can help to differentiate it. Neonates and infants who are suspicious of MSUD should never be challenged with higher than normal protein intake for diagnostic purposes.

#### Scenario 2: symptomatic patients

These individuals either did not perform NBS, or the NBS result was falsely negative, or confirmatory testing was not done despite abnormal NBS. In such instances, some clinical or biochemical findings may suggest MSUD in these patients. Supportive clinical findings include the following: In untreated infants, it consists of Maple syrup odor in the secretions, signs of encephalopathy including intermittent apnea, lethargy, opisthotonus, and stereotyped movements such as ‘bicycling” and “fencing”. Central respiratory failure and coma may occur from seven to ten days of age. Untreated older individuals with milder variants of MSUD may present with manifestations such as anorexia, poor growth, irritability, developmental delay, and acute attacks of hyperleucinemia, ketonuria, and encephalopathy triggered by fasting, dehydration, surgery, or infections.

Laboratory findings for these patients included: Elevated plasma BCAAs and alloisoleucine; Urinary excretion of BCKDs and branched-chain alpha-ketoacid (BCKAs) in infants older than 48–72 h; Ketonuria detected by standard urine test strips; Hypoglycemia and/or hyperammonemia without definite cause [5, 11].

Confirmation of Diagnosis in an MSUD suspicious patient is confirmed by the identification of biallelic pathogenic variants in one of the genes or limited cases by considerably decreased activity of the BCKD enzyme in leukocytes, cultured fibroblasts, or biopsied liver tissue. Molecular genetic testing is the preferred confirmatory test for MSUD.

In the abnormal newborn screening (NBS) results in favor of MSUD, the preferred molecular genetic test is using a multigene panel that contains *BCKDHA*, *BCKDHB*, and *DBT*. In the symptomatic patients with clinical findings suggestive of MSUD, If the biochemical result is highly suggestive of MSUD, the molecular genetic testing approach is the same as the situation mentioned item. But if the biochemical lab is not highly suggestive of MSUD, and is not considered, whole exome sequencing is a better option and most commonly used [5].

### Treatment

Every patient who is highly suspicious of MSUD according to plasma amino acid levels should be treated immediately. Management of the patients should not be delayed while awaiting the molecular genetic result [12].

#### Treatment of acute metabolic crisis

Metabolic decompensation episodes in patients should be treated quickly. The principle goal of treatment in these patients is the reduction of the leucine level by 500 to 1000 µmoles/L/day [13]. Symptomatic patients should preferably be admitted to the PICU. In case of presenting with nausea or vomiting, decreased oral intake, fever, diarrhea, lethargy, or neurological symptoms including seizures, changes in level of consciousness, ataxia, dystonia, and headache, evaluation should be started immediately. Initial laboratory evaluation includes the following:

- Chemistry panel for sodium, bicarbonate levels, and anion gap (to evaluate for increased anion gap metabolic acidosis and hyponatremia).
- Venous blood gas.
- Plasma amino acids profile.
- Urine ketone.
- Workup for infections (CBC, cultures, chest X-ray, urinalysis, etc.) [14].

The general principles of treatment include the following:

1. Stop natural protein intake
2. Hydration and calorie supply
3. Correcting metabolic abnormalities
4. Toxin removal
5. Finding the underlying cause of the metabolic crisis
6. Cofactor treatment
7. Minimizing complications [15]

Natural Protein intake should be stopped for 24 to 48 h [13, 16]. This period could be extended to 24 to 72 h in adults [15]. We recommend 1.25–1.5 times intravenous maintenance. Fluids should be given as 10–12.5% dextrose in normal saline [14–16].

The amount of dextrose can be increased to 25% in patients with central vein catheters. Intralipid solution can be used with a dose of 1–2 g/kg/day to provide more calories [10, 13].

It is necessary to check blood sugar every 4 to 6 h. The goal is to maintain blood sugar between 100 and 160 mg/dl [17]. In case of hyperglycemia, insulin infusion can be started at the rate of 0.02–0.15 IU/Kg/h, which helps to improve anabolism [14, 17, 18].

Serum sodium measurement is recommended every 12 to 24 h. Serum sodium levels should be maintained between 138 and 145 mEq/L. To reach the desired sodium level, it is sometimes necessary to give a NaCl supplement [16]. If possible, check the osmolality level and maintain it between 275 and 300 mOsm/kg H_2_O and avoid serum osmolality from decreasing > 5 mOsm/kg H_2_O per day [17].

Protein as BCAA-free amino acids with a dose of 2–3.5 g/ kg /day (if the intravenous form is available) together with isoleucine and valine supplements (20 to 120 mg/kg/day titrate to plasma level remains between 400 and 800 µmole/L) were recommended for leucine reduction [16, 17, 19]. For patients that can tolerate oral feeding, continuous feeding of BCAA-free MSUD formula with an NG tube in the range of 30 to 60 cc per hour (0.7–1.2 kcal/ml) should be started [19].

Dialysis should be considered in the case of leucine of more than 1100 µmole/L and/or rapid onset of neurological symptoms [21]. The use of short-course continuous hemodialysis is more effective than peritoneal dialysis and veno-venous hemofiltration [22]. It should be noted that dialysis without treatment of underlying disease and nutritional support is not effective [23].

Sodium phenylbutyrate reduces BCAA and can be effective in patients with intermediate MSUD. However, its effectiveness is controversial and more studies are needed [12, 24]. Triggering factors including infection, trauma, and dehydration should be treated. A low clinical threshold for empiric administration of antibiotics when signs of infection are present should be considered. Control of fever with antipyretic and control of nausea and vomiting with ondansetron (0.15 mg/kg/dose) is recommended. Candida infection is common in these patients.

Drugs such as Ketorolac (in cases of dehydration, kidney disease, and use of drugs that affect renal perfusion) are contraindicated, and glucocorticoids and vasoactive catecholaminergic agents are limited in these patients [17].

**Cofactor therapy in these patients is useful.** Some patients are treated with thiamine as thiamine responsive with a dose of 50–200 mg daily, in these patients, thiamine should be given orally with the previous dose, and in case of oral intolerance, it should be given intravenously [25–29].

laboratory monitoring includes the following:

- Blood sugar every 4 to 6 h with a glucometer.
- Blood amino acids, serum phosphate, and magnesium every 12 to 24 h [17].
- Amino acid check should be done in a laboratory that can prepare results within 24 h (if applicable) [15].
- Lipase, amylase, and transaminases every 24 to 48 h [17].
- Blood sodium every 12 to 24 h [17].
- Urine ketones with each urination [14].

If ketones are not clearing, this likely means that the patient is not receiving adequate nutrition and calories, or that the underlying stressor is significantly preventing anabolism [14]. The most common biochemical complications in patients are hyperglycemia, hypoglycemia, hyponatremia, hypokalemia, and hypophosphatemia [17].

#### Complications

1- *Cerebral edema*: When intracranial hypertension occurs, the patient should be admitted to the ICU and neurological consultation should be done. Symptomatic hypo-osmolality or worsening signs of intracranial hypertension should be treated with the following options including mannitol 0.5–1 g/kg/dose, hypertonic (3%) saline: 2–3 mEq/kg/dose and furosemide 0.5-1 mg/kg/dose. In cases of moderate to severe encephalopathy, we start NaCl drip 5 to 15 mEq/kg/day, to the extent that serum osmolality is maintained between 290 and 300 mOsm/kg H_2_O, serum sodium is maintained in the range of 138 to 145 mEq/L, and serum osmolality change below 5 mOsm/kg H_2_O per day [9]. For obtunded patients, consider endotracheal intubation and neurosurgical consultation [17]. Brain imaging is indicated in certain cases. In some cases, with unstable conditions, a CT scan of the brain is performed as MRI could not be done in unstable patients [17].

2-*Acute pancreatitis*: The patient should be NPO, measure lipase and amylase, and start supportive treatment with an intravenous solution without branched chain amino acids [20].

3-*Dystonia and acute crisis*: oral tyrosine should be used 100 to 400 mg/kg/day [17].

Management of the acute crisis of MSUD in hospitals is shown in Table 2.

**Table 2:** Management of Acute Crisis of MSUD in Hospital

Intervention	Detail
Diagnostic Intervention^*^	- Laboratory tests: CBC diff, ESR, CRP, BUN, creatinine, Na, K, VBG, Ca, Ph, AST, ALT, ALP, ammonia, lactate, blood culture, urine culture - Chest X-ray (if required).
Therapeutic Intervention^**^	
Generic	- IV fluid DW10% (1/25 * maintenance) + NaCl 150 mEq/L + KCl 20 mEq/L - Natural Protein intake should be stopped for 24 to 48 h. BCAA free amino acids with a dose of 2–3.5 g/ kg /day can be recommended as the patient tolerates. - TPN (R/O infection); initiate intralipid 1gr/kg to reach 2 g/kg. - Electrolytes and blood sugar should be kept within the normal range during treatment; 100 < BS < 160 mg/dl, 138 < Na < 140 mq/L, 275 < serum osmolarity < 300 osm/kg H_2_O. - Avoid taking drugs such as Ketorolac, glucocorticoids, and catecholaminergic agents.
Specific	- Clinical brain edema; Manito 0/25–1 g/kg infusion for 20–30 min, or Saline 3% 5 cc/kg for 15 min - Peritoneal dialysis or hemodialysis indication; Luciene > 1100, severe brain edema, retractile seizure, opisthotonos, decreased consciousness, and hypotonia resistant to treatment. - In thiamine responsive MSUD, administer thiamine 50–200 mg/day. - Dystonia and acute crisis: Oral tyrosine should be used 100 to 400 mg/kg/day.

*Blood sugar every 4 to 6 h with a glucometer; Blood amino acids, serum phosphate, and magnesium every 12 to 24 h; Amino acid check should be done in a laboratory that can prepare results within 24 h; Lipase, amylase, and transaminases every 24 to 48 h should be checked; Blood sodium every 12 to 24 h should be checked; Urine ketones with each urination should be evaluated

** The principal goal of treatment in these patients is a reduction of the leucine level by 500 to 1000 µmol/L daily

### Sick day management

During fever, surgery, or any infectious disease or stress, there is a possibility of a sudden increase in leucine, acidosis, hypoglycemia, and a decrease in consciousness. In this situation, sometimes the damage to the neurological system may be irreversible. For this reason, it is necessary to take correct and appropriate actions quickly. The sick day protocol should be started as soon as appearing of the first symptoms of the disease. This protocol is applicable only for minor diseases that can be treated at home.

Mild cases of increased catabolism that can be treated on an outpatient basis include fever under 38.5 °C, tolerance of oral feeding or NG tube feeding, without frequent vomiting or severe diarrhea, and absence of neurological symptoms such as consciousness changes, restlessness, and hypotonia. Otherwise, the patient needs hospitalization and intravenous treatment. The treatment of hospitalized cases is given in the previous part of this article.

Necessary measures during illness include:

- Inform the healthcare provider and ask for guidance.
- Refer to a pediatric specialist to check the cause of the disease and the required treatment if necessary.
- The consumption of leucine should be reduced by 50–100%.
- The consumption of natural protein should be reduced or stopped.
- The consumption of BCAA-free formula should be increased to the 120% of the usual consumption.
- Increasing the consumption of isoleucine and valine supplements (20–40 mg/kg/day of each).
- Frequently consuming liquids containing glucose or other forms of carbohydrates such as apple juice, grape syrup, and high-glucose drinks.
- Consuming pure carbohydrate powders such as carbomeal^®^ or carbomass^®^ to provide the calories needed by the child.
- Periodic measurement of urine BCAA using dinitrophenyl hydrazine (DNPH) strips (if available) or urine test for ketones (acetoacetate). Because the elimination of ketosis is a guide for the adequacy of fluid and energy replacement.
- Re-evaluation of clinical changes every two hours in terms of changes in consciousness and food tolerance and the need to go to the hospital or contact the metabolic center.
- Monitoring BCAA level in plasma or blood every 24 to 48 h to guide the proper adjustment of the diet during the disease (To be decided according to the available facilities).
- Gradual consumption of natural protein after about 48 h.
- Prescribing acetaminophen or ibuprofen to reduce fever if necessary.

### Monitoring

It should be noted that the goal of the treatment is not to eliminate BCAAs but to keep them in an appropriate range so that sufficient mental and physical growth can happen. Therefore, the patients should be on a diet their whole life. Leucine level should never be above 1.5 to 2 times the normal range.

The acceptable levels of BCAAs are as follows:

- Plasma *Leucine* level: children ≤ 5 years of age: 75–200 µmol/L and children > 5 years of age: 75–300 µmol/L.
- Plasma valine and isoleucine levels: 200 to 400 µmol/L [12, 24].
- Some specialists advise that valine levels should be at least twofold leucine levels and isoleucine levels should be equal to leucine levels.

To note, valine and isoleucine deficiency can lead to acrodermatitis enteropathica [30]. The intellectual outcome is related to the leucine level in the first 6 years of life [31]. However, leucine tolerance increases with age.

In stable patients, monitoring of blood or plasma BCAA is done every 1 to 2 weeks until 5 years of age, then every 2 to 4 weeks until 12 years of age, and monthly afterward [32]. In cases where it is difficult to access the laboratory or the results of the tests are prepared with a delay, the doctor may increase the test intervals according to the patient’s condition. However, it should be noted that increasing the test intervals may cause problems in controlling the disease. Isoleucine and valine supplements are added to the diet at the discretion of the physicians. Thiamine should be continued in thiamine-responsive patients [33]. Essential supplements such as Fe, zinc, calcium, selenium, vitamin A, and vitamin D should be given to the patients.

Plasma levels of amino acid, albumin, CBC, Methylmalonic acid (MMA), Homocysteine, Ferritin, Zinc, vitamin D, and biochemistry should be checked regularly.

Liver transplant is promising and leads to normal diet toleration. Due to the various side effects, it is only done in patients who cannot be controlled with diet and have multiple attacks. Before the transplant, patients’ metabolic conditions must be stable with no decompensation attacks. The addition of Branched-Chain Amino Acids (BCAAs) should be carefully and gradually implemented [34, 35].

### Diet (medical nutrition management)

Every infant with MSUD should be hospitalized at the beginning of treatment so that his/her clinical evaluation is done completely his/her tolerance to nutritional recommendations is evaluated and parents are well educated about nutrition and the course of treatment. The primary goals in treating MSUD are to manage diet by:

1. Reducing BCAAs (Limited high protein foods and the diet is supplemented with leucine, valine, and isoleucine-free l‐amino acids).
2. Provide adequate macronutrients to prevent catabolism.
3. Maintain plasma BCAAs within targeted treatment ranges.
4. Additional valine and isoleucine supplements are invariably necessary.

To ensure adequate protein synthesis, energy intake should meet the estimated average requirements for age [36] (Table 3). The dietary protein restriction is guided by leucine requirements in Table 3. The need for leucine is usually met by using breast milk or infant formula. Leucine from breast milk or infant formula is replaced by solid foods when the infant is developmentally ready [37–40].

**Table 3:** Recommended daily nutrient intake of BCAA, PRO, Energy, and fluids for non-symptomatic individuals with MSUD

Age	LEU (mg/kg)	ILE (mg/kg)	VAL (mg/kg)	Protein (g/kg)	Energy (kcal/kg)	Fluid (mL/kg)
0–6 months	40–100	30–90	40–95	2.5–3.5	95–145	125–160
7–12 months	40–75	30–70	30–80	2.5–3.0	80–135	125–145
1–3 years	40–70	20–70	30–70	1.5–2.5	80–130	115–135
4–8 years	35–65	20–30	30–50	1.3–2.0	50–120	90–115
9–13 years	30–60	20–30	25–40	1.2–1.8	40–90	70–90
14–18 years	15–50	10–30	15–30	1.2–1.8	35–70	40–60
19 years	15–50	10–30	15–30	1.1–1.7	35–45	40–50

#### Nutritional management of newly diagnosed infants with MSUD

In infants, the goal is rapid leucine reduction to achieve target treatment levels. Breastfeeding (BF) and infant formula (leucine source) are stopped temporarily. Oral or Enteral feeding should be commenced early. Provide 3 g/kg/day BCAA-free protein equivalent. A high-energy formula (120–140 kcal (500–585 kJ)/kg/ day) to promote anabolism. Glucose polymer is usually added to BCAA‐free infant formula to provide a total of 10 g CHO/100mL. The fat emulsion is added, as necessary. Isoleucine and valine (varying between 200 and 400 mg/day) to support maximal rates of protein synthesis [15] Leucine is given from standard infant formula/ expressed breastmilk (EBM) when plasma leucine is < 800 µmol/L. As leucine intake from infant formula increases, this natural protein will also provide a source of isoleucine and valine, so these individual supplements should be reduced and adjusted to maintain them within target treatment reference ranges (Monitoring biochemical section).

A total protein intake of 3 g/kg/day from standard infant formula and BCAA-free infant formula is necessary. Once oral feeding is established, the BCAA‐free infant formula and infant formula should be administered in separate feeding bottles. A measured volume of infant formula is given (6–8 times/day) followed by BCAA‐free infant formula. An infant with MSUD can be breastfed. During initial stabilization, BCAA‐free infant formula and valine and isoleucine supplements are given until leucine levels are < 800 µmol/L, with the mother expressing breastmilk. Infants can then be breastfed on demand, but always with a measured quantity of BCAA‐free infant formula first, with the volume titrated according to BCAA amino acid concentrations. There are few reports of BF infants with MSUD [41].

#### Introduction of solids. Nutrients to infants with MSUD

In such patients, solids should be introduced around 17–26 weeks of age starting with 1–2 teaspoons of natural low protein exchange-free foods such as homemade purée fruits and vegetables, e.g. apple, pear, carrot, butternut squash, and parsnip [42]. Low protein weaning foods are usually offered after the breast or formula feeds, so it does not alter the volume of breast milk/formula consumed. The intake of BCAA‐free infant formula should be carefully monitored to ensure the amount is adequate. At this stage, the low protein food is mainly offered for the infant to establish a taste for solid foods, and they usually accept these without difficulty [43]. Many low-protein weaning foods have a low energy density, so higher-energy weaning foods should be encouraged. Weaned foods are gradually increased to three times a day. Once infants are taking 8–12 teaspoons at a time, natural protein exchange foods are given instead of the equivalent quantity of infant formula or breastfeeding. 1 g natural protein exchange should be introduced, gradually replacing all breast or formula feeds with the equivalent natural protein source from solid food. More textured food should be gradually introduced from 6 to 8 months.

#### Non-classic MSUD dietetic management

The management required for this group of patients varies based on the type of MSUD. For intermediate MSUD, leucine restriction ± branched chain free amino acids and an ER are necessary. For intermittent MSUD, may only require moderate protein restriction (no branched chain free amino acids) and an ER. Also, in thiamin-responsive phenotype patients, leucine restriction ± branched chain free amino acids and thiamin supplementation and an ER; thiamin is required during illness, fasting, infection, or surgery [44].

In E3-deficiency patients, a BCAA‐restricted diet does not reverse or prevent ongoing symptoms, but can maintain blood BCAA concentrations within target ranges; ER is necessary.

#### Complications of nutritional treatment in MSUD

- Decreased height for age may be observed [45].
- Higher BMI at the age of 5 and 10 years in children with MSUD compared with other IMDs treated with low protein diets was observed. Energy intake in the early treatment years was > 200% of the predicted basal metabolic rate (BMR), contributing to long-term increased BMI and % fat mass [46].
- Nutritional deficiencies may occur including valine and isoleucine, n-3 fatty acids [23], and selenium. Chronic deficiency of BCAA may cause anemia, acrodermatitis, hair loss, growth failure, arrested head growth, anorexia, and lethargy [24, 47].
- Low bone mineral density (BMD) is also reported in adolescents. Bone fractures cause transient leucosis.

The items of dietary treatment are provided in Table 4.

**Table 4:** Emergency regimens in MSUD

Diet	Guidance
BCAA-free amino acid substitute to support protein synthesis	Daily dose 0–3 years: 3 g/kg protein equivalent 4–5 years: 2.5 g/kg protein equivalent 6–10 years: 2 g/kg protein equivalent 11–14 years: 1.5 g/kg protein equivalent 15–16 years: 60 g/day protein equivalent
Valine and isoleucine supplementation	
Glucose polymer supplement (concentration for age)	Up to 1 (years) 10% 1–2 (years) 15% 2–9 (years) 20% > 10 (years) 25%
Fat source	If fat is tolerated, the fat concentration could be 3–5 g per 100 mL of emergency feed.
Natural protein intake (leucine exchanges)	Stop or reduce by at least 50% (depending on the severity of illness and BCAA results
Introduction to dietary leucine post-illness	Leucine < 800 µmoles/L: 25% usual leucine intake Leucine > 400 and < 600 µmoles/L: 50% usual leucine intake Leucine < 400 µmoles/L: 75% of usual intake Repeat leucine < 400 µmoles/L: Usual leucine intake

## Summary

MSUD is a rare inherited metabolic disorder with a diverse presentation and clinical progression. Establishing guidelines for patient care is a pivotal aspect of the healthcare system, crucial for effective long-term management and treatment outcomes. Given the challenges of limited access and high costs associated with laboratory testing, treatment, and patient follow-up for MSUD in various countries, particularly in developing nations, the development of the guideline with restricted accessibility was essential. The challenges in the management and screening of MSUD faced in Iran are mentioned. It is also presented to better manage patients in acute metabolic crises, sick days, and nutritional treatment in such circumstances. Finally, this hopefully provides tailored guidance to other physicians in countries with similar limited resources.

## Data Availability

Not applicable.

## Abbreviations

BCAA: Branched-chain amino acid
BCKDH: Branched-chain 2-ketoacid dehydrogenase complex
BF: Breastfeeding
BMD: Bone mineral density
BMR: Basal metabolic rate
DLD: Dihydrolipoamide dehydrogenase
EBM: Expressed breastmilk
MMA: Methylmalonic acid
MSUD: Maple syrup urine disease
NBS: Newborn screening
